# Turning Up the Heat: Local Temperature Control During *in vivo* Imaging of Immune Cells

**DOI:** 10.3389/fimmu.2019.02036

**Published:** 2019-08-27

**Authors:** David Ahl, Olle Eriksson, John Sedin, Cédric Seignez, Emil Schwan, Johan Kreuger, Gustaf Christoffersson, Mia Phillipson

**Affiliations:** Department of Medical Cell Biology, Uppsala University, Uppsala, Sweden

**Keywords:** intravital microscopy, skin, blood flow, leukocytes, vacuum window, confocal microscopy

## Abstract

Intravital imaging is an invaluable tool for studying the expanding range of immune cell functions. Only *in vivo* can the complex and dynamic behavior of leukocytes and their interactions with their natural microenvironment be observed and quantified. While the capabilities of high-speed, high-resolution confocal and multiphoton microscopes are well-documented and steadily improving, other crucial hardware required for intravital imaging is often developed in-house and less commonly published in detail. In this report, we describe a low-cost, multipurpose, and tissue-stabilizing *in vivo* imaging platform that enables sensing and regulation of local tissue temperature. The effect of tissue temperature on local blood flow and leukocyte migration is demonstrated in muscle and skin. Two different models of vacuum windows are described in this report, however, the design of the vacuum window can easily be adapted to fit different organs and tissues.

## Introduction

Microscopy has a long history as an important tool in life science research. In recent decades, more advanced microscope technologies have been steadily emerging, providing researchers with a plethora of options for visualization of complex biological processes. Intravital microscopy provides unique information about cellular behavior and functions within the living animal. With the invention of the confocal microscope (1) and its ability to optically section the imaged tissue, the possibility of imaging cell-cell interactions *in vivo* was greatly improved. Further technological advances allowed for the introduction of the multiphoton microscope, which improved some of the limiting aspects of confocal microscopy, such as phototoxicity and penetration depth. While these microscopy techniques enable high temporal and spatial resolution imaging, other aspects of *in vivo* imaging remain challenging, and impact the quality of the results. The access to and preparation of the target tissue should enable maintained regulation of homeostasis including stable blood perfusion, innervation and temperature, which otherwise will influence the observed biological processes. In addition, stabilization of the target tissue is crucial for high-resolution imaging, and even very slight movements can prevent acquisition of usable image data. When performing imaging in anesthetized laboratory animals, the breathing of the animal is the most common cause of movement artifacts, also in tissues distal to the lungs. Devices using vacuum for immobilization of the lungs and other organs have been previously described, but has required custom-made metallic parts to function, somewhat increasing the threshold for most researchers to acquire such systems (2–5).

Maintaining proper physiological conditions while exposing the tissue to the microscope objective is important if the benefits of studying biological processes *in vivo* are to be retained. While standard practice is to carefully control the body temperature of the animal with a whole-body heating pad, local tissue temperature of the imaged area is rarely reported. Attaching the tissue to any kind of stabilizing device or imaging window may further increase the risk of heat loss when the ambient-tempered device is in physical contact with the tissue and may act as a heat-sink. Interestingly, the importance of local temperature control when studying immune cell behavior was recently highlighted in a paper by Lin et al. (6) demonstrating that T-cell trafficking and bacterial clearance were affected by febrile temperatures.

In an effort to overcome these challenges, we here utilize 3D printing together with commercially available electronic components to produce a versatile and low-cost *in vivo* imaging platform. The platform consists of a temperature-regulated vacuum window, a vacuum window holder and an operating table with magnetic mounting points. We also present two proof-of-concept applications for the platform: The study of blood flow using heat-induced hyperemia and the effect of local tissue temperature on leukocyte migration.

## Materials and Methods

### 3D-Printing

All 3D-printed parts were designed in Fusion 360 (Autodesk Inc., San Rafael, CA, USA). The vacuum window holder and the vacuum windows were printed with a Form 2 3D-printer (Formlabs, Somerville, MA, USA) with 50 μm layers. The vacuum window holder was printed in clear resin (Formlabs). The vacuum windows were printed in Dental SG resin (Formlabs) and post-processed according to Formlabs guidelines to reach biocompatibility. The operating table was 3D-printed in black PLA (polylactic acid) with an Ultimaker 3 Extended 3D printer (Ultimaker B.V., Geldermalsen, The Netherlands) using a 0.8 mm nozzle and 200 μm layers with the ironing feature (Neosanding) enabled when preparing toolpaths in Ultimaker Cura 4.0.0 (Ultimaker). All STL files are available in the Supplementary Data.

### Magnets

The magnets inserted in the base of the operating table and the vacuum window holder were neodymium disk magnets, of N42 grade with nickel plating and a diameter of 15 mm and a height of 8 mm (Webcraft GmbH, Gottmadingen, Germany).

### Electronic Components

The electronic control unit was built using readily available standard components. Specifically, an Arduino UNO Rev3 microcontroller was connected to a MOSFET IRF520 module (Velleman, Gavere, Belgium), a 100 kΩ NTC bead thermistor (NXFT15WF104FA2B100, Murata Electronics, North America) and a 32 AWG nichrome resistance wire.

### Animals

Male C57Bl/6J mice [30–35 g (Taconic, Denmark)], male CX_3_CR1^GFP/+^/NG2DsRedBAC F1 hybrid mice [25–30 g [B6.129P-Cx3cr1tm1Litt/J, The Jackson Laboratory, (7)] and (Tg(Cspg4-DsRed.T1)1Akik, The Jackson Laboratory, (8)] were used. All animals had access to tap water and pelleted food *ad libitum* throughout the experimental study. For blood flow measurements, fur was removed by shaving the right hind limb of the mice 1 day prior to the experiments. For intravital imaging, the following antibodies were administered through an injection in the tail vein approximately 30 min before start of imaging to visualize leukocytes and blood vessels: anti-mouse Ly6G mAb conjugated to Brilliant Violet 421, anti-mouse CD31 mAb conjugated to Alexa Flour Dye 647. All experiments were approved by the Regional Animal Ethics Committee in Uppsala, Sweden, under the ethical permit number C81/14.

### Surgical Preparation, Blood Flow Measurements, IR, and Intravital Imaging

Mice were anesthetized by spontaneous inhalation of isoflurane (Abbott Scandinavia, Sweden) diluted 1.8–2.6 % in a mixture of air and oxygen. The animals were immediately placed on the operating table described in this article, with a heating pad to control and maintain body temperature. IR temperature imaging was performed using a FLIR A40 thermal imaging camera and the FLIR Report Studio software (FLIR, Wilsonville, OR, USA). For the blood flow measurements, the left jugular vein was catheterized with a PE-10 cannula for administration of L-NAME and saline, and the right carotid artery was catheterized and connected to a BP transducer (MLT0380/D) and a Bridge Amplifier (ML221). Blood pressure data was recorded at 1 kHz sampling rate by a PowerLab 4/3 and analyzed in LabChart 7 Pro software (AD Instruments, Oxford, UK). Blood flow was measured using Laser Speckle Contrast Analysis (785 nm laser, 20 μm resolution, PeriCam HR PSI System, Perimed). For intravital imaging, a ~10 × 10 mm patch of the abdominal skin was carefully dissected from the mouse and turned upside down, creating a skin flap still connected to the mouse abdomen. The vacuum window, described in this article, was covered with an imaging-grade 13 mm No. 1.5 glass coverslip held in place with vacuum grease, where after the inside of the mouse abdominal skin was carefully immobilized using the vacuum. Prior to attachment, the coverslip had been prepared with a 0.5 mm^3^ agarose gel (2% in Hanks balanced salt solution, Sigma-Aldrich) containing MIP-2 (0.5μM), (R&D Systems) to induce chemotaxis of leukocytes.

The table was then mounted on a Scientifica MMBP stage connected to a Leica SP8 point-scanning confocal microscope with a Leica HC Fluotar L 25 ×/0.95-W VISIR objective. Experiments were conducted in a room temperature of 23.5±°C.

### Analysis

Data is presented as mean ± SEM and data analysis was performed using GraphPad Prism 6 (GraphPad Software, La Jolla, CA, USA). Student's two-tailed *t*-test was used analyzing two groups and *p* < 0.05 was considered significant.

Intravital time-lapse movies were processed using the Fiji distribution of ImageJ and the TrackMate plugin (9–11).

## Results

The purpose of the vacuum window imaging platform is to immobilize tissue from an anesthetized animal to enable *in vivo* high resolution imaging, primarily using an upright confocal or multi photon microscope. In addition, maintained tissue temperature is essential when studying biological parameters. To allow for this, as well as to enable the study of the effects of locally altered temperature, the vacuum window described here was designed to provide both regulation and readout of tissue temperature.

**Figure 1:**
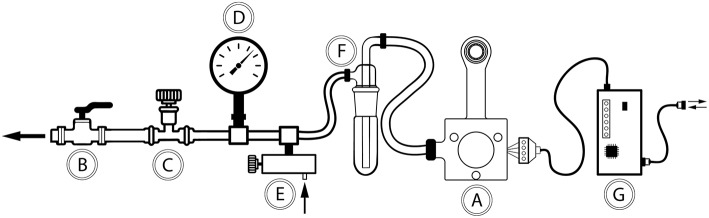
Schematic drawing of the components in the vacuum window *in vivo* imaging platform. **(A)** Vacuum window, **(B)** connection to central vacuum system (ON/OFF), **(C)** coarse vacuum adjustment valve, **(D)** negative pressure gauge, **(E)** vacuum wastegate for fine tuning of negative pressure, **(F)** vacuum trap, **(G)** Arduino microcontroller.

At the center of the *in vivo* imaging platform is a 3D-printed vacuum window (Figure 1A). The window was connected to a vacuum source (Figure 1B). The negative pressure was regulated through a coarse adjustment valve (Figure 1C) and fine-tuned using a negative pressure gauge (Figure 1D) and a manually regulated vacuum wastegate (Figure 1E). A vacuum trap (Figure 1F) was used to protect the vacuum source. Further, the vacuum window was connected to an Arduino microcontroller (Figure 1G) through a 4-wire cable, enabling reading of temperature data as well as controlling temperature through a heating wire inside the vacuum window.

### Temperature-Regulated Vacuum Window

**Figure 2:**
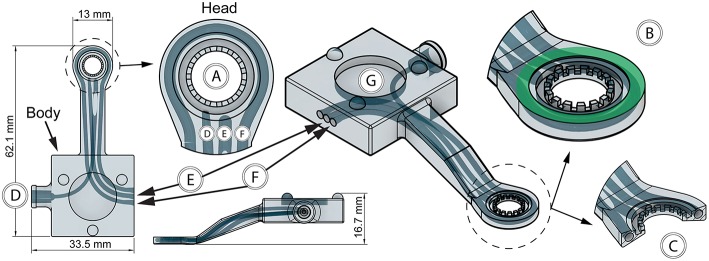
Features of the 3D-printed vacuum window. **(A)** Window opening, **(B)** coverslip attachment surface (green), **(C)** vacuum window head cross section, **(D)** vacuum channel, **(E)** thermistor channel, **(F)** heating wire channel, **(G)** steel plate cutout for kinematic mount.

To enable imaging of tissue, the vacuum window was designed with a circular opening in the head of the window (Figure 2A). Importantly, the flat top of the 13 mm vacuum window head serves as the attachment surface for a round glass coverslip (Figure 2B, green area), which is hermetically sealed to the window frame using a thin layer of vacuum grease. The vacuum window head was constructed so that it together with the coverslip constitutes the upper half of a vacuum connected chamber (Figure 2C). When vacuum is applied through the vacuum channel (Figure 2D), tissue positioned below the vacuum window head will be pulled upward and held against the coverslip, enabling imaging through the coverslip. In our experiments, 5–20 mbar was enough negative pressure to get stable attachment of the tissue to the coverslip.

To enable registration of temperature, a thermistor channel (Figure 2E) was positioned in the vacuum window, allowing entry of the thermistor wires in the vacuum window body and positioning of the thermistor head as close to the window opening as possible. This channel was designed to also have an opening close to the head of the window to facilitate insertion of the thermistor during assembly. Heat delivery to the window was provided by a 32 AWG nichrome resistance wire inserted in a heating channel in the vacuum window (Figure 2F). The heating channel openings were positioned on both sides of the thermistor channel in the vacuum window body, while the channel itself circumvents the vacuum window opening in the head to enable heat transfer to the imaged area when a current is applied to the resistance wire. During assembly, the nichrome wire was first inserted into a PTFE (OD/ID: 0.9 mm/0.4 mm) tube to facilitate insertion into to the heating channel. After assembly of both the thermistor and heating wire, all channel openings were sealed with a cyanoacrylate adhesive. To allow for magnetic attachment to the holder, the top of the vacuum window body was designed with a cutout for a round steel plate (15 × 2 mm) that was glued in place during assembly (Figure 2G).

The vacuum window was 3D-printed using a biocompatible resin using a Form 2 printer. STL files for printing the window can be found in the Supplementary File 1. Notably, the vacuum window design can easily be adapted to fit a specific organ of interest.

### Electronic Control Unit

**Figure 3:**
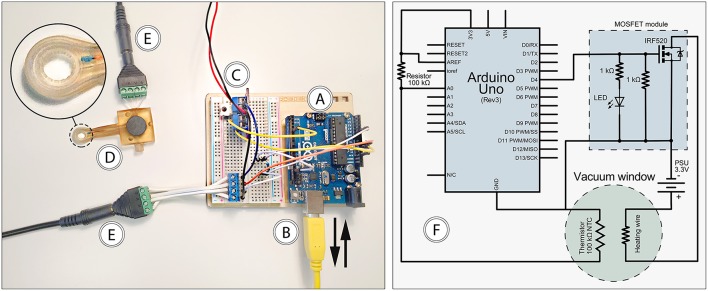
Features of the electronic control unit. **(A)** Arduino Uno, **(B)** USB connection to computer, **(C)** 3.3 V power supply and MOSFET module, **(D)** vacuum window, **(E)** 4-wire cable, **(F)** circuit diagram.

For measuring and regulating tissue temperature, a control unit was built using an Arduino Uno Rev3 microcontroller (Figure 3A) powered by a USB cable connected to a computer (Figure 3B). The USB interface was also used for logging thermistor data and setting target temperature. Heating wire power was provided through a MOSFET module connected to a standard computer power supply unit delivering 3.3 V (Figure 3C). The vacuum window (Figure 3D) was connected to the control unit using a 4-wire cable (Figure 3E) with screw terminal connectors in each end to attach the four wires.

The computer connected to the control unit through the USB interface (Figure 3B) was running the Arduino IDE software, which provided continuous logging of thermistor temperature as well as setting target temperature for the control loop. The control loop was designed so that it reads the temperature of the thermistor, compares it with the set target temperature, and adjusts power output to the heating wire using a PID regulator. ON/OFF functionality of the heating system was achieved with the power switch of the power supply unit. A complete circuit diagram of the control unit is provided in Figure 3F. The code for the microcontroller can be found in the Supplementary File 2.

### Holder and Operating Table

In order to achieve stable immobilization of tissue attached to the vacuum window, a vacuum window holder (to be mounted on a steel plate) and an operating table with magnet attachment points was designed and 3D-printed. STL and Gcode files for printing the holder and operating table can be found in the Supplementary File 1.

**Figure 4:**
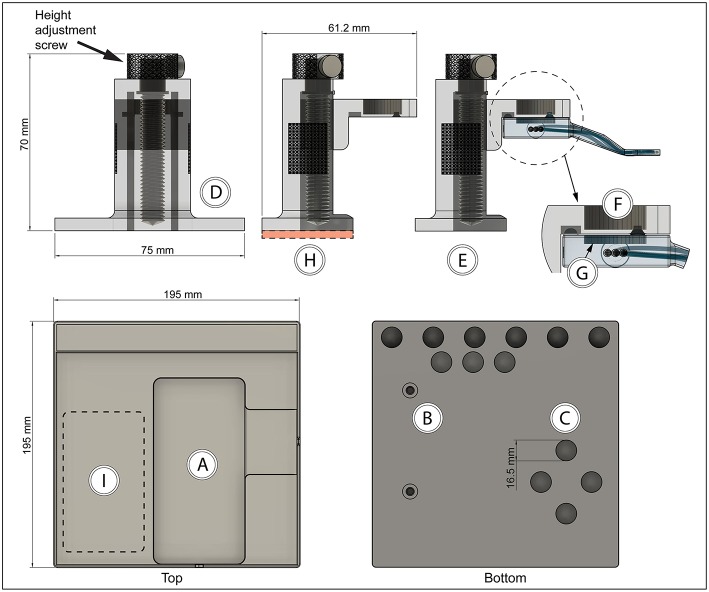
Vacuum window holder and operating table with magnetic attachment points **(A)** Heating pad cutout, **(B)** M5 thread inserts, **(C)** neodymium magnet cutouts, **(D)** height-adjustable vacuum window holder—front view, **(E)** holder side view with vacuum window attached, **(F)** neodymium magnet position on holder, **(G)** steel plate position in vacuum window, **(H)** height-adjustable vacuum window holder—side view, red area indicates position of steel plate, **(I)** holder attachment area on table.

The operating table (printed in PLA using an Ultimaker 3 Extended 3D printer) had cutouts for a heating pad (Figure 4A), M5 thread inserts for mounting of the table on a Scientifica stage (Figure 4B), and 15 mm round neodymium magnets (Figure 4C). Thread inserts and magnets were glued in place during assembly.

The vacuum window holder was printed in clear resin using a Form 2 printer. After assembly, the height adjustment screw in the base of the holder (Figure 4D) can be used to position the vacuum window vertically (Figure 4E). The mounting part of the holder consists of a kinematic mount that fits to the vacuum window body. Attachment of the window to the holder is achieved using a neodymium magnet (Figure 4F) on the mount and a steel plate in the vacuum window (Figures 2G, 4G).

**Figure 5:**
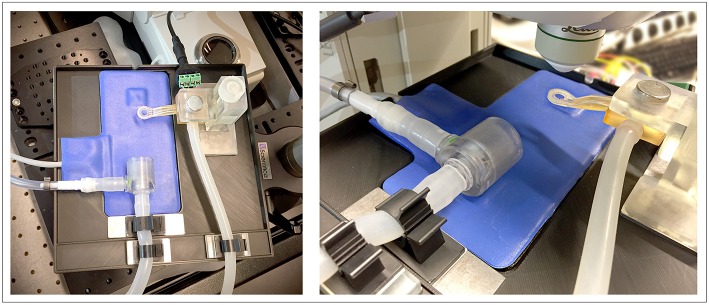
Complete setup of the 3D-printed *in vivo* imaging platform mounted on a Scientifica stage on the Leica SP8 confocal microscope, shown together with heating pad, anesthetic mask and metal plate mounted holders for tubing.

The height-adjustable holder was mounted on a 2 mm steel plate (Figure 4H, red area). Together with the magnetic attachment points on the operating table and the vacuum window, this constitutes a stable tissue immobilizing platform while at the same time providing great flexibility in XYZ positioning of the imaging window (Figure 4I). The neodymium magnets also function as attachment points for any other steel plate mounted peripherals, such as holders for tubing (Figure 5).

Figure 5 depicts the complete setup mounted with additional magnetically attached holders for tubing on a Scientifica stage for imaging with an upright Leica SP8 confocal microscope.

### *In vivo* Temperature Control Dynamics

The thermistor embedded in the vacuum window head was positioned as close as possible to the underlying tissue. However, during heating, the temperature in window opening is expected to be lower due to the distance from the heating wire circumventing the window. In order to elucidate the relationship between the temperature reported by the thermistor and the temperature inside the window viewing area below the coverslip, a set of experiments was performed using a FLIR A40 thermal imaging camera. By setting up temperature measuring points in the acquired IR images using the FLIR Report Studio software, temperature readouts in positions inside and surrounding the viewing area were recorded.

**Figure 6:**
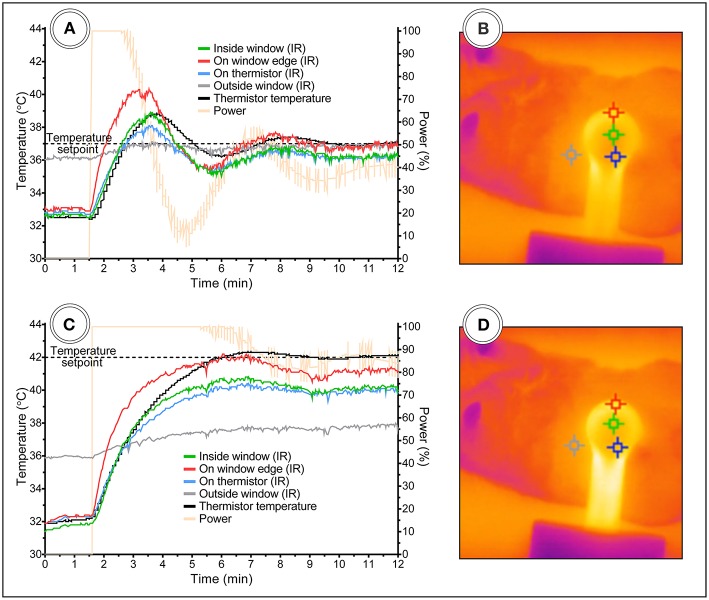
Vacuum window temperature control dynamics in anesthetized mice with skin flap attached to window. Temperature setpoint at **(A)** 37°C and **(C)** 42°C. **(B,D)** Shows the corresponding IR images with temperature measurement points.

The dynamics of local tissue temperature regulation *in vivo* was tested using two different temperature setpoints with the vacuum window attached to the inside of an abdominal skin flap of an anesthetized C57Bl/6J mouse. First, a physiological tissue temperature of 37°C was used as the setpoint for the temperature control loop. As shown in Figure 6A, an overshoot, most pronounced at the window edge containing the heating wire (Figure 6A, red line), up to 40°C was briefly reached, followed by an undershoot. After 10 min, a stable temperature of approximately 36°C was reached in the center of the imaging window. The positions of the IR temperature measurement points are shown in Figure 6B.

Secondly, a supraphysiological tissue temperature of 42°C was set for the control loop (Figure 6C). This setpoint did not produce an overshoot, most likely due to the fact that it appeared to be close to the maximum temperature possible with a 3.3 V power source. This is supported by the power readout (Figure 6C, yellow line) from the control unit, showing that maximum power was delivered for the first 5 min, after which power started to be reduced by the control loop. After 10 min, the IR temperature reading inside the window stabilized at approximately 40°C. The positions of the IR temperature measurement points are shown in Figure 6D.

In these experiments, both the embedded thermistor and the IR camera reported that the initial tissue temperature inside the window was lower than physiological temperature at around 32–33°C, while the shaved skin of the mouse outside of the window maintained approximately 36°C (Figures 6A–D, gray line and marker), indicating that the vacuum window serves as a heat-sink when the heating is turned off and room temperature is lower than skin temperature.

### L-NAME Attenuates Heat-Induced Hyperemia in Mouse Hind Limb Skin and Underlying Muscle

**Figure 7:**
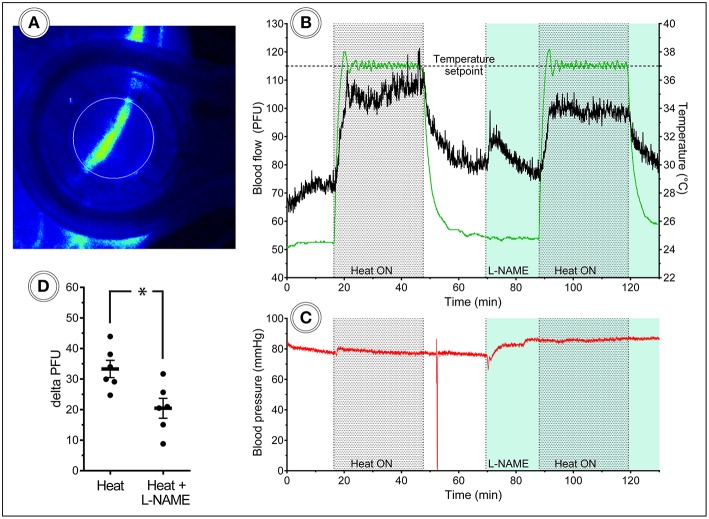
Laser speckle blood flow imaging in the mouse hind limb. **(A)** Laser speckle contrast image of a mouse hind limb with heat turned off, **(B)** 130 min continuous blood flow and **(C)** arterial blood pressure measurements, including two heat challenges and L-NAME administration, **(D)** heat-induced hyperemia is attenuated after L-NAME administration (*n* = 6).

Given the ability of the vacuum window presented here to manipulate local tissue temperature, we wanted to test this function in an experimentally relevant setting; the study of heat-induced hyperemia. Heat-induced hyperemia can be used to study functional blood flow regulation, and has previously been achieved by applying heat to an entire limb through tubing containing pre warmed water to increase skin temperature 10°C. The temperature-regulated vacuum window developed here could provide researchers with a tool for more controlled experimental settings, including more precise readout of local tissue temperature. To this end we conducted a set of experiments in anesthetized C57Bl/6J mice using a Laser Speckle Contrast Analysis system, which provides quantitative image data of tissue blood flow. Fur was removed from the hind limb of mice 1 day prior to the day of the experiment. Blood flow was measured with the vacuum window head placed on the exposed skin of the hind limb, and the window opening was selected as the measurement area (Figure 7A). Continuous blood flow measurements were performed for 130 min during which the vacuum window heat was turned on with a setpoint of 37°C for two separate periods of 20 min each. Nitric oxide produced by the endothelial nitric oxide synthase (eNOS) is known to regulate blood flow (12, 13). The L-arginine analog L-NAME (N(ω)-nitro-L-arginine methyl ester) is a widely used eNOS inhibitor (14), thereby affecting the ability of vasculature to dilate. After the first heating and a cool-down period of 20 min, a bolus dose (10 mg/kg) of L-NAME was administered, after which continuous infusion (3 mg/ml at 0.35 ml/h) of L-NAME was initiated (Figure 7B). The mean blood flow (perfusion units) during 15 min before each heating cycle was considered as the baseline value of blood flow, whereas the plateau phase of the blood flow curve during the heating was considered the hyperemic blood flow value. The difference (delta PFU) between baseline and hyperemia provides a readout of the ability of the animal to regulate blood flow in response to heat. Arterial blood pressure was monitored throughout the experiments (Figure 7C). Heating the vacuum window to 37°C produced a marked increase in local blood flow, whereas L-NAME attenuated this increase (Figure 7D). These results demonstrate the possibility of using the temperature-regulated vacuum window as a tool for studying functional blood flow.

### Leukocyte Migration Speed at Different Tissue Temperatures

The main purpose of the temperature-regulated vacuum window is to provide researchers with a tissue stabilizing tool for *in vivo* imaging that gives the experimenter control over local tissue temperature. To investigate the effects of different tissue temperatures on leukocyte migration, we conducted experiments in anesthetized mice with fluorescently labeled leukocytes.

Neutrophils were immunostained with Ly6G mAb conjugated to Brilliant Violet 421 in CX_3_CR1^GFP/+^/NG2DsRedBAC mice, where GFP is expressed by CX_3_CR1^+^ cells (predominantly monocytes, macrophages and dendritic cells), and DsRed marks vascular mural cells (pericytes) to provide more detailed information about the vascular morphology. In the imaging data, pericytes (red) can be seen covering arterial vasculature and as smaller dots along capillaries, but are absent on the venous vasculature. Blood vessels were immunostained with CD31 mAb conjugated to Alexa Flour Dye 647.

**Figure 8:**
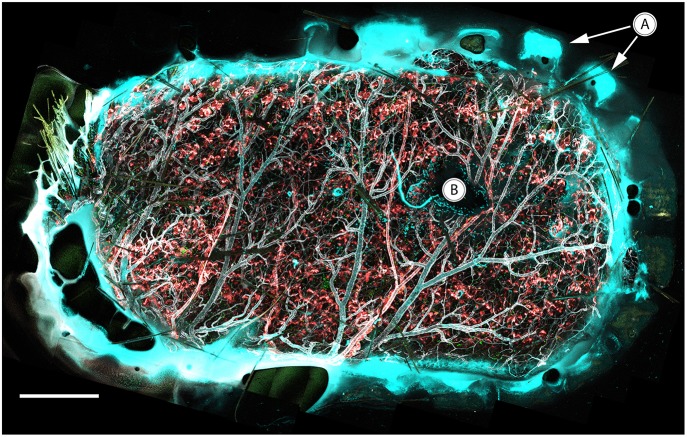
*In vivo* confocal tile scan of the complete vacuum window opening attached to immunostained mouse skin. **(A)** Teeth of the window border lining the tissue, **(B)** MIP-2 gel positioned between the coverslip and underlying tissue. Color code: Ly6G–cyan, CX_3_CR1–green, CD31–gray, NG2–red. Scale bar is 400 μm.

To induce leukocyte recruitment from the vasculature, an agarose gel containing MIP-2 was attached to the coverslip of the vacuum window before it was lowered onto the inside of an abdominal skin flap. The appearance of immunostained tissue attached to the vacuum window coverslip is demonstrated in Figure 8, showing a tile scan that covers the complete window opening of the oval vacuum window. The teeth of the inner window edge are visible (Figure 8A) and mark the border between the mouse skin and the plastic material of the window. The MIP-2 gel is also visible as a dark shape blocking light from the stained tissue (Figure 8B). A Leica SP8 confocal microscope was used to acquire time-lapse recordings of the migrating leukocytes while the vacuum window was cycled ON-OFF in 20 min intervals with a supraphysiological temperature setpoint of 42°C.

**Figure 9:**
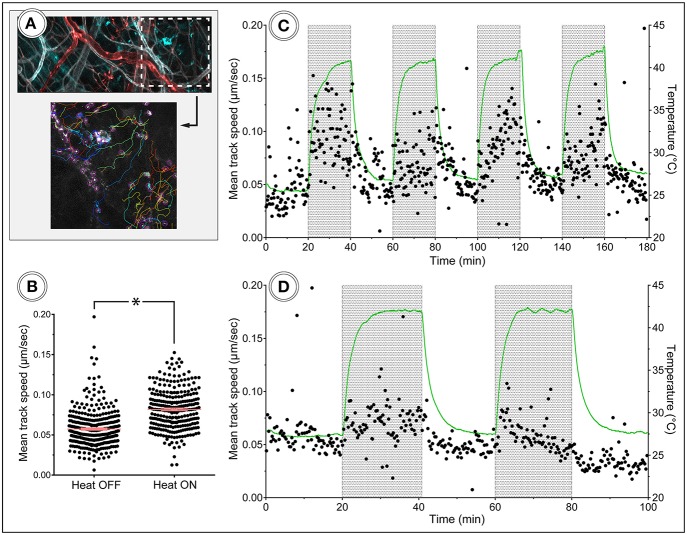
*In vivo* tracking of leukocyte migration during heat challenge. **(A)** Shows a frame from the corresponding time-lapse movie and tracking data. Color code: Ly6G–cyan, CD31–gray, NG2–red. **(B)** Migration speed of neutrophils was increased during heating periods compared to periods without heating. **(C,D)** Show track speed of neutrophils and CX_3_CR1 positive cells, respectively, plotted over time with heating periods indicated by gray areas.

Leukocytes were tracked using the TrackMate plugin for ImageJ (Figure 9A), and the speed of the cells was plotted for the duration of the experiments. The tracking of cells in these data sets was performed so that only migrating cells were included in the analysis. Time-lapse movies and images visualizing cell tracking from the experiments are available in the Supplementary Files 3–7. Neutrophil migration speed was increased during the periods of heating (Figures 9B,C), while migrating CX_3_CR1 cells showed a less clear response to heat (Figure 9D), indicating that temperature control is of importance when doing intravital imaging of leukocytes. The NG2-positive cells were not visibly affected by the heat-cycling in these experiments.

## Discussion

Here we present the most complete and easily accessible solution to date for enabling *in vivo* imaging of virtually any organ of anesthetized mice. This was achieved by further development of previous designs (3, 4) of vacuum windows to be able to monitor and control temperature of the imaged tissue. The vacuum window was designed with a magnetic mounting system that makes pre-experimental set up of the platform easy.

The readily available and low-cost electronic components together with the supplementary code for the Arduino microcontroller provides the researcher with an easily assembled bare-bones control unit. While the specific electronic parts used in this paper will produce an easy-to-use functional system, most parts can be substituted with alternative components, e.g., in order to achieve a more compact setup and fit it in a small chassis. Addition of fuses and diodes can also easily be made to provide short-circuit protection if required. To further facilitate ease of use of the system, we chose to include a complete operating table in the platform. The neodymium magnets embedded in the bottom of the table provide an attachment area where the vacuum window holder, or any other steel-mounted peripheral device, can be freely positioned while still maintaining excellent stability. This gives the platform great flexibility, allowing researchers to use the temperature-regulated vacuum window at any position of the anesthetized mouse suitable for their specific application. In addition, magnets embedded in the top edge of the table allows for attachment of steel-mounted holders to secure tubing for vacuum and anesthesia. The emergence of low-cost, commercially available 3D printers has led to massive growth of the 3D printing market (15). The scientific community has embraced the opportunity to create custom-built laboratory tools for a wide range of applications (16–19). The *in vivo* imaging platform described in this paper was 3D printed, and all STL files are available for download in order to ease use of the design.

The test of the temperature regulating ability of the vacuum window demonstrated that it is possible to attain stable temperature using both physiological and supraphysiological setpoints within 10 min of operation. Presumably, this time period can be shortened if the vacuum window is pre-heated instead of starting from ambient temperature. Of note is also that without heating turned on, the temperature reported by the IR measurement point inside of the viewing area was significantly lower (32–33°C) than that of the shaved skin of the mouse (36°C). This indicates that the window itself might be acting as a heat sink, lowering temperature in the observed tissue, further emphasizing the importance of being able to control temperature in the vacuum window in order to maintain physiological conditions during imaging. These experiments also provide insight in the relationship between thermistor temperature and tissue temperature. At a temperature setpoint of 37°C, tissue temperature was approximately one degree lower than the thermistor reading, and at a 42°C setpoint, tissue temperature was two degrees lower. We believe that this is due to the fact that the thermistor is positioned closer to the heating wire than to the center of the vacuum window opening, which leads to poorer heat transfer from the wire to the tissue in the center of the window than to the thermistor. Thus, with heating activated, heat transfer becomes a relevant factor which results in 3–5% lower tissue temperature compared to the setpoint of the vacuum window. This difference appears to increase with setpoint temperature. If the temperature regulating function of the vacuum window is to be utilized, and uncertainty below 5% is required, we suggest that the researcher investigates the temperature discrepancy between the thermistor and the center of the vacuum window with the tissue of interest attached to the window.

In order to compare the performance of the temperature-regulated vacuum window to established methods, we conducted a set of experiments on blood flow regulation in the mouse hind limb. These experiments test the ability of the mouse vasculature to up regulate blood flow in response to a local heat increase. Our results show that the vacuum window successfully induces hyperemia without affecting arterial blood pressure, and that administration of L-NAME attenuates this response. Compared to the conventional method of using tubing with pre-heated water, the vacuum window provides a simpler and more effective way of achieving hyperemia, with the added benefit of more precise temperature regulation due to the embedded thermistor.

Tracking migrating cells *in vivo* is associated with several challenges related to the preparation and stabilization of tissue. *Ex vivo* solutions such as explanting lymph nodes or spleens for leukocyte imaging are commonly used to circumvent this, with the drawbacks of disconnecting blood circulation with risk of hypoxia, and removing the organ from its original position and thus losing the possibility of leukocyte trafficking in and out of the organ. Other solutions for *in vivo* imaging may include extracorporeal tissue fixation to a viewing pedestal with the possibility of heat and fluid dissipation. For true physiological readouts, intact tissue within the animal must be handled in such a way so that sufficient blood flow is maintained in order to avoid ischemia, and any mean of stabilizing the tissue must be gentle enough not to cause unnecessary trauma that might affect cell behavior in the imaged area. In our proof-of-concept *in vivo* imaging experiments, the temperature-regulated vacuum window fulfilled the function as a tissue-stabilizing device eliminating artifacts such as those emanating from respiratory movements. When heat was cycled to a supraphysiological setpoint of 42°C to mimic fever, the increased migration speed of neutrophils was apparent, both visually and according to tracking data. CX_3_CR1 positive cells showed a much less pronounced migration speed response to heat, indicating that all leukocytes do not respond the same to fever.

Taken together, the present study provides researchers with details on a 3D printed, low-cost *in vivo* imaging platform with a temperature-regulated vacuum window. The presented proof-of-concept experiments demonstrate the function of the vacuum window as a tissue stabilizer as well as the importance of local temperature regulation when performing *in vivo* imaging of leukocytes.

## Data Availability

The datasets generated for this study are available on request to the corresponding author.

## Author Contributions

DA and CS conducted the experiments. DA, JS, OE, JK, ES, GC, and MP designed the method and study. DA, GC, and MP analyzed the data and interpreted the results. DA wrote the manuscript. DA, OE, JK, ES, GC, and MP contributed to the editing of the manuscript.

### Conflict of Interest Statement

The authors declare that the research was conducted in the absence of any commercial or financial relationships that could be construed as a potential conflict of interest.

## Supplementary Material

Supplementary Video 1 180 minute *in vivo* time-lapse movie from the Leica SP8 confocal microscope, imaging mouse skin attached to the vacuum window. Neutrophils can be seen migrating during heat-cycling of the vacuum window. Color code: Ly6G–cyan (neutrophils), CD31–gray (blood vessels), NG2–red (pericytes).

Supplementary Video 2 100 minute *in vivo* time-lapse movie from the Leica SP8 confocal microscope, imaging mouse skin attached to the vacuum window. CX_3_CR1^+^-cells can be seen migrating during heat-cycling of the vacuum window. Color code: CX_3_CR1–green (monocytes/macrophages/dendritic cells), CD31–gray (blood vessels), NG2–red (pericytes).

Supplementary Images 1–3 Screenshots from visualization of neutrophil tracking in Supplementary Video 1 using the TrackMate plugin for ImageJ. Color code: Ly6G–gray (neutrophils), tracks are colorized to facilitate identification of individual tracks.
