# Supplementary material for: Turning Up the Heat: Local Temperature Control During in vivo Imaging of Immune Cells
Source: Front Immunol. 2019 Aug 27;10:2036. doi: 10.3389/fimmu.2019.02036 (PMC6718468; doi:10.3389/fimmu.2019.02036)
Supplement: Supplementary file 2 [file Table_1.DOCX]

//include EEPROM

#include <EEPROM.h>

//EEPROM map

//bytes 0 - 3 float P

//bytes 4 - 7 float I

//bytes 8 - 11 float D

//bytes 12 - 15 float targetTemp

//bytes 16 - 19 float B constant

//bytes 20 - 23 float Rt resistance of thermistor

//bytes 24 - 27 float Rr resistance of resistor

//bytes 28 - 31 float Ot thermistor calibration offset

//include for PID-library

#include <PID_v1.h> //http://playground.arduino.cc/Code/PIDLibrary, https://github.com/br3ttb/Arduino-PID-Library

//include for parsing serial command

#include <SerialCommand.h> //https://github.com/scogswell/ArduinoSerialCommand/

// Pin assignments

#define outputPin 3 //Output pin for heat power

#define thermistorPin A1 //Pin for thermistor

#define ledPin 13 //Pin for led

#define numTermReads 10 //number of termistor reads for a value

//Structure for commands

struct cmd {

byte c; //command character, T=target temp, W=wait in seconds, D=disable temp control

double v; //command value

};

//Vars for temp

double targetTemp;

double currentTemp;

double output = 0;

double readValue = 0;

bool TempNotRead = true;

bool heatingEnabled=false; //is heating on

//Vars for PID

double Kp;

double Ki;

double Kd;

double B;

double Rt;

double Rr;

double Ot;

//Vars for command sequence

cmd seq[16]; //Array to hold sequence

int seqpos; //Current position in sequence

int seqlength; //lenth of command sequence

bool seqEnabled=false; //is sequence enabled

//Var for timings

unsigned long heatPulseStart=0; // time of last heatpulse start

unsigned long heatDutyOff=0; //time when heat duty cycle ends and heater shuts off

unsigned long commandDone=0; //time when the current command has finished

//Time settings

#define heatPulse 500 //time of heat pulse cycle in ms

//Specify the links and initial tuning parameters

PID myPID(&currentTemp, &output, &targetTemp, Kp, Ki, Kd, DIRECT);

//Set up Serial command

SerialCommand swCmd;

void setup()

{

//Set outputpin and LED pin to ouput

pinMode(outputPin, OUTPUT); //output pin

pinMode(ledPin,OUTPUT); //LED pin

//initialise serial over USB

Serial.begin(115200);

while (!Serial);

//Set up serial commands for software serial

swCmd.addCommand("T",Tcmd); //set Target Temp and enable direct temperature control

swCmd.addCommand("P",Pcmd); //set P in PID

swCmd.addCommand("I",Icmd); //set I in PID

swCmd.addCommand("D",Dcmd); //set D in PID

swCmd.addCommand("B",Bcmd); //set B constant for thermistor

swCmd.addCommand("Rt",Rtcmd); //set resistance of thermistor

swCmd.addCommand("Rr",Rrcmd); //set resistance of resistor connected to thermistor

swCmd.addCommand("Ot",Otcmd); //set thermistor calibration offset

swCmd.addCommand("val",valcmd); //request print of values

swCmd.addCommand("add",addcmd); //add command to end of sequence "add T 37.2"

swCmd.addCommand("delseq",delseqcmd); //delete teh entire sequence

swCmd.addCommand("seq",seqcmd); //request print of command sequence

swCmd.addCommand("seqon",seqoncmd); //enable sequence command control disable direct temperature control

swCmd.addCommand("off",offcmd); //disable sequence, disable heating

swCmd.addDefaultHandler(unrecognizedCmd);

//Read from EEPROM

EEPROM.get(0, Kp);

EEPROM.get(4, Ki);

EEPROM.get(8, Kd);

EEPROM.get(12, targetTemp);

EEPROM.get(16, B);

EEPROM.get(20, Rt);

EEPROM.get(24, Rr);

EEPROM.get(28, Ot);

PrintStat();

//initialise PID library

myPID.SetMode(AUTOMATIC);

myPID.SetOutputLimits(0.0,100.0);

myPID.SetTunings(Kp, Ki, Kd);

delay(1000);

}

void loop() {

//About 100 ms before heatpulse start reading temp

if (millis()>heatPulseStart+heatPulse-100 && TempNotRead) {

ReadTemp();

TempNotRead=false;

}

//Output heatpulse and calculate

if (millis()>heatPulseStart+heatPulse) {

myPID.Compute();

PrintCurr();

heatPulseStart=millis();

heatDutyOff=map(int(output*100),0,10000,heatPulseStart,heatPulseStart+heatPulse);

TempNotRead=true;

if (int(output)>0) {

digitalWrite(outputPin, HIGH);

digitalWrite(13,HIGH);

}

}

//Output off

if (millis()>heatDutyOff) {

digitalWrite(outputPin,LOW);

digitalWrite(13,LOW);

}

//Read serial command

swCmd.readSerial();

//handle sequence control

if (seqEnabled && millis()>=commandDone) {

seqpos+=1;

if (seqpos>seqlength) {seqpos=1;}

Serial.print("Sequence position: ");

Serial.print(seqpos);

switch (seq[seqpos-1].c) {

case 'T':

targetTemp=seq[seqpos-1].v;

heatingEnabled=true;

myPID.SetMode(AUTOMATIC);

Serial.print(" Set target temp to: ");

Serial.println(targetTemp);

break;

case 'W':

commandDone=commandDone+seq[seqpos-1].v*1000;

if (commandDone<=millis()) {commandDone=millis()+seq[seqpos-1].v*1000;}

Serial.print(" Wait until: ");

Serial.println(commandDone);

break;

case 'D':

heatingEnabled=false;

myPID.SetMode(MANUAL);

output=0;

Serial.println(" Heating disabled.");

break;

default:

Serial.print("Unrecognised command in sequence: ");

Serial.println(seq[seqpos-1].c);

break;

}

}

}

void PrintCurr() {

Serial.print("Time: ");

Serial.print(millis());

Serial.print(" T: ");

Serial.print(currentTemp);

Serial.print(" Target: ");

if (heatingEnabled) {Serial.print(targetTemp);} else {Serial.print("off");}

Serial.print(" Out: ");

Serial.println(int(output*100));

}

void PrintStat() {

Serial.print("Kp ");

Serial.println(Kp);

Serial.print("Ki ");

Serial.println(Ki);

Serial.print("Kd ");

Serial.println(Kd);

Serial.print("target ");

Serial.println(targetTemp);

Serial.print("B ");

Serial.println(B);

Serial.print("Rt ");

Serial.println(Rt);

Serial.print("Rr ");

Serial.println(Rr);

Serial.print("Ot ");

Serial.println(Ot);

Serial.print("Heat ");

Serial.println(heatingEnabled?"Enabled":"Disabled");

Serial.print("SeqCmd ");

Serial.println(seqEnabled?"Enabled":"Disabled");

}

void ReadTemp() {

int value = 0;

for(int i = 0;i < numTermReads;i++) {

value += analogRead(thermistorPin);

delay(10);

}

readValue = value / numTermReads;

// https://learn.adafruit.com/thermistor/using-a-thermistor

double steinhart = 1023 / readValue - 1;

steinhart = Rr / steinhart;

steinhart = steinhart / Rt;

steinhart = log(steinhart);

steinhart /= B;

steinhart += 1.0 / (25.0+273.15);

steinhart = 1.0 / steinhart;

steinhart -= 273.15;

steinhart += Ot;

currentTemp = steinhart;

}

//Commands received

void Tcmd() {

char *arg;

arg = swCmd.next();

if (arg != NULL) {

targetTemp=atof(arg);

seqEnabled=false;

heatingEnabled=true;

myPID.SetMode(AUTOMATIC);

PrintStat();

EEPROM.put(12,targetTemp);

} else {

Serial.println("T: No data.");

}

}

void Pcmd() {

char *arg;

arg = swCmd.next();

if (arg != NULL) {

Kp=atof(arg);

PrintStat();

myPID.SetTunings(Kp, Ki, Kd);

EEPROM.put(0,Kp);

} else {

Serial.println("P: No data.");

}

}

void Icmd() {

char *arg;

arg = swCmd.next();

if (arg != NULL) {

Ki=atof(arg);

PrintStat();

myPID.SetTunings(Kp, Ki, Kd);

EEPROM.put(4,Ki);

} else {

Serial.println("I: No data.");

}

}

void Dcmd() {

char *arg;

arg = swCmd.next();

if (arg != NULL) {

Kd=atof(arg);

PrintStat();

myPID.SetTunings(Kp, Ki, Kd);

EEPROM.put(8,Kd);

} else {

Serial.println("D: No data.");

}

}

void Bcmd() {

char *arg;

arg = swCmd.next();

if (arg != NULL) {

B=atof(arg);

PrintStat();

EEPROM.put(16,B);

} else {

Serial.println("B: No data.");

}

}

void Rtcmd() {

char *arg;

arg = swCmd.next();

if (arg != NULL) {

Rt=atof(arg);

PrintStat();

EEPROM.put(20,Rt);

} else {

Serial.println("Rt: No data.");

}

}

void Rrcmd() {

char *arg;

arg = swCmd.next();

if (arg != NULL) {

Rr=atof(arg);

PrintStat();

EEPROM.put(24,Rr);

} else {

Serial.println("Rr: No data.");

}

}

void Otcmd() {

char *arg;

arg = swCmd.next();

if (arg != NULL) {

Ot=atof(arg);

PrintStat();

EEPROM.put(28,Ot);

} else {

Serial.println("Ot: No data.");

}

}

void valcmd() {

PrintStat();

}

bool isseqcmd(char str[]) {

bool test=false;

if (strcmp(str, "T")==0) {test=true;}

if (strcmp(str, "W")==0) {test=true;}

if (strcmp(str, "D")==0) {test=true;}

return test;

}

void printSeq(int s) {

Serial.print(s);

Serial.print(' ');

Serial.print((char)seq[s-1].c);

Serial.print(' ');

Serial.println(seq[s-1].v);

}

void seqcmd() {

Serial.println("Current sequence:");

for (int i=1;i<=seqlength;i++) {

printSeq(i);

}

if (seqEnabled) {

Serial.print("Current sequence position: ");

Serial.println(seqpos);

Serial.print("Command change in ");

Serial.print(commandDone-millis());

Serial.println(" ms.");

} else {

Serial.println("Sequence control disabled");

}

}

void seqoncmd() {

seqEnabled=true;

seqpos=0;

commandDone=millis();

Serial.println("Sequenced control enabled. Sequence started from beginning.");

}

void offcmd() {

seqpos=0;

seqEnabled=false;

heatingEnabled=false;

myPID.SetMode(MANUAL);

output=0;

Serial.println("Heating off, sequence control off.");

}

void addcmd(){

char *arg;

arg = swCmd.next();

Serial.println(arg);

if (isseqcmd(arg)) {

seq[seqlength].c=arg[0];

arg = swCmd.next();

Serial.println(arg);

if (arg != NULL)

{

seq[seqlength].v=atof(arg);

} else {

seq[seqlength].v=0;

}

seqlength+=1;

printSeq(seqlength);

} else {

Serial.println("Command not recognised");

}

}

void delseqcmd() {

offcmd();

for (int i=0;i<16;i++) {

seq[i].c=NULL;

seq[i].v=NULL;

}

seqlength=0;

Serial.println("Sequence deleted");

}

void unrecognizedCmd() {

Serial.println("Command unrecognised.");

}
