# Supplementary figures and images for: Turning Up the Heat: Local Temperature Control During in vivo Imaging of Immune Cells
Source: Front Immunol. 2019 Aug 27;10:2036. doi: 10.3389/fimmu.2019.02036 (PMC6718468; doi:10.3389/fimmu.2019.02036)

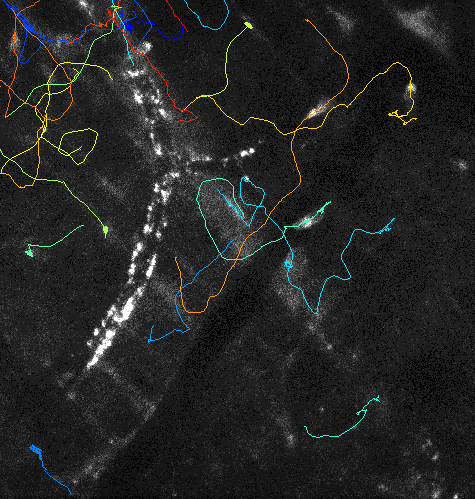

Supplement: Supplementary file 5 [file Image_1.TIF]

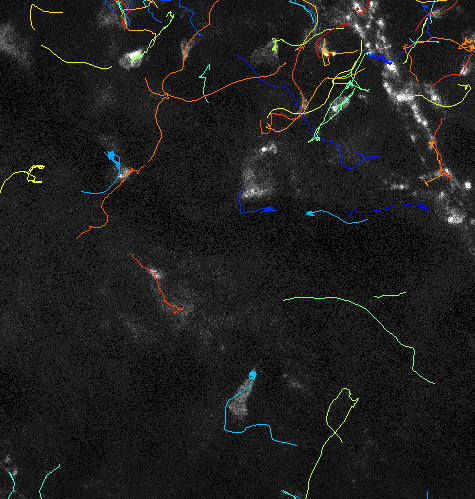

Supplement: Supplementary file 6 [file Image_2.TIF]

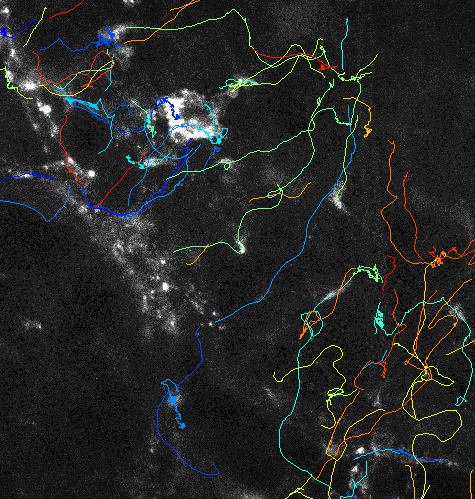

Supplement: Supplementary Images 1–3 — Screenshots from visualization of neutrophil tracking in Supplementary Video 1 using the TrackMate plugin for ImageJ. Color code: Ly6G–gray (neutrophils), tracks are colorized to facilitate identification of individual tracks. [file Image_3.TIF]
